# Positive Allosteric Modulation of mGluR5 Accelerates Extinction Learning but Not Relearning Following Methamphetamine Self-Administration

**DOI:** 10.3389/fphar.2012.00194

**Published:** 2012-11-26

**Authors:** Peter R. Kufahl, Lauren E. Hood, Natali E. Nemirovsky, Piroska Barabas, Casey Halstengard, Angel Villa, Elisabeth Moore, Lucas R. Watterson, M. Foster Olive

**Affiliations:** ^1^Department of Psychology, Arizona State UniversityTempe, AZ, USA

**Keywords:** mGluR5, cognitive enhancement, extinction learning, methamphetamine, reinstatement, positive allosteric modulator

## Abstract

Recent studies have implicated glutamate neurotransmission as an important substrate for the extinction of conditioned behaviors, including responding for drug reinforcement. Positive allosteric modulation of the type-5 metabotropic glutamate receptor (mGluR5) in particular has emerged as a treatment strategy for the enhancement of extinction of drug-motivated behaviors. Here, we investigated the effects of the mGluR5 positive allosteric modulator CDPPB, a compound known for its cognitive enhancing effects in rodents, on extinction learning in rats with different histories of methamphetamine (METH) training. Rats were trained to self-administer METH under two conditions: 16 daily sessions of short access (90 min/day, ShA), or eight daily sessions of short access followed by eight sessions of long access (6 h/day, LgA). Control rats self-administered sucrose pellets in daily 30 min sessions. Next, rats were administered vehicle or 30 mg/kg CDPPB prior to seven consecutive daily extinction sessions, subjected to additional extinction sessions to re-establish a post-treatment baseline, and then tested for reinstatement of behavior in the presence of METH- or sucrose-paired cues. Rats were then subjected to a second series of extinction sessions, preceded by vehicle or 30 mg/kg CDPPB, and an additional test for cue-triggered reinstatement. CDPPB treatment resulted in a more rapid extinction of responding on the active lever, especially in the early sessions of the first extinction sequence. However, treatment effects were minimal during subsequent cue reinstatement tests and non-existent during the second series of extinction sessions. Rats with histories of ShA, LgA, and sucrose training expressed similar behavioral sensitivities to CDPPB, with LgA rats demonstrating a modestly higher treatment effect. Positive allosteric modulation of mGluR5 may therefore have some beneficial effects on efforts to facilitate extinction learning and reduce methamphetamine seeking.

## Introduction

Addiction to methamphetamine (METH) is marked by continued use in spite of adverse consequences, as well as chronic relapse to drug-taking after extended periods of abstinence. Relapse behaviors are preceded by strong feelings of drug craving, which are triggered by sensory cues that activate powerful memory traces of past drug experiences (O’Brien et al., 1998; Barr et al., 2006). The motivational salience of drugs such as METH is a consequence of direct activation of neurobiological reward systems, engaging and overpowering the regulatory systems that normally regulate the response to food and other non-drug rewards (Di Chiara and Bassareo, 2007). In vulnerable individuals, experimental or casual METH taking brings about maladaptive changes in the brain beyond the reward circuitry, involving systems associated with learning and memory functions (Childress et al., 1999; Volkow et al., 2008; Koob and Volkow, 2010). Accumulating evidence of these alterations in preclinical studies and human brain neuroimaging experiments has led to the conceptualization of drug addiction as a disorder of learning and memory systems (Kelley, 2004; Hyman et al., 2006). Though several models of addiction as a learning disorder have been proposed, they tend to share the common argument that the progression of the disease is characterized by two forms of aberrant learning: drug-associated environmental cues attaining, through a Pavlovian conditioning process, a persistent incentive salience capable of triggering craving, and relapse behaviors (associative overlearning), and drug-taking behavior growing into a compulsive habit (instrumental overlearning, also a form of associative learning; Volkow et al., 2002; Ciccocioppo et al., 2004).

The contributions of instrumental and associative overlearning to drug-motivated behavior are often studied in rats utilizing some variant of the extinction-reinstatement model, where animals are trained to self-administer drug reinforcers in the presence of cues, subjected to repeated sessions of extinction training until responding is decreased to a certain criterion, and then reintroduced to the drug-paired cues and tested for the reinstatement of behavior pursuant to drug delivery (Shaham et al., 2003; Sanchis-Segura and Spanagel, 2006). With this technique, METH-seeking behavior has been tested under the influence of various compounds that represent candidate neurobiological targets for anti-relapse therapeutics (Kufahl and Olive, 2011). However, the mechanisms underlying the reduction of drug-taking operant behavior during extinction training have been examined far less frequently (Taylor et al., 2009). Extinction of self-reported METH craving has been shown in METH-dependent individuals via cue exposure sessions in a laboratory setting (Price et al., 2010), and incorporation of repeated sessions of unreinforced cue exposures has been proposed in the treatment of addiction to drugs in general (O’Brien et al., 1990). However, this methodology has proven to have limited benefits toward the prevention of relapse and related behaviors in rats (Crombag and Shaham, 2002) and humans (Conklin and Tiffany, 2002). While many explanations of the limitations of extinction training have been proposed, what seems clear is that this approach may benefit from a thorough understanding of the neurochemical substrates of this learning process, and introduction of a pharmacological treatment that enhances extinction performance and prolongs its effect on subsequent behavior (Myers and Carlezon, 2010).

Glutamate neurotransmission is a major component of long-term potentiation and long-term depression of synaptic transmission, which are core cellular processes of learning and memory (Byrne, 2008). Multiple experiments utilizing the extinction-reinstatement model have associated dysregulated glutamate release and receptor function with the reinstatement of operant responding for drugs triggered by cues, drug priming, and stress (Gass and Olive, 2008). Potentiation of NMDA receptor function by the partial agonist d-cycloserine has been found to improve performance of various learning tasks in rodents (Monahan et al., 1989; Richardson et al., 2004) including the extinction of previously established cocaine place preference (Botreau et al., 2006; Thanos et al., 2009) and operant responding for cocaine reinforcement (Nic Dhonnchadha et al., 2010; Torregrossa et al., 2010; Thanos et al., 2011). Additionally, the cysteine prodrug *N*-acetylcysteine, by stabilizing extracellular glutamate levels via the cystine/glutamate exchange pump, has been shown to enhance extinction of responding for drug reinforcement and protect against subsequent reinstatement (Zhou and Kalivas, 2008; Moussawi et al., 2011). Adding to the promise of targeting glutamate function is the growing evidence linking glutamate release and the direct and conditioned rewarding effects of METH (Fujio et al., 2005; Nakagawa et al., 2005; Osborne and Olive, 2008). Long-lasting imbalances in synaptic and extrasynaptic glutamate are now compellingly associated with the loss of control over drug-seeking behaviors (Kalivas, 2009; Kalivas et al., 2009).

Glutamate neurotransmission is highly regulated through a complex system of receptors, intracellular pathways, exchangers, transporters, and other mechanisms (Niciu et al., 2012), and targeting modulatory metabotropic glutamate receptors (mGluRs) is increasingly preferred over targeting ionotropic glutamate receptors (iGluRs) as a therapeutic strategy associated with a favorable profile of potential side effects (Uys and LaLumiere, 2008; Olive, 2009). Exerting control over glutamate release by manipulation of mGluRs has become a primary focus of efforts to find long-lasting reductions in addictive behaviors (Knackstedt and Kalivas, 2009). MGluR_5_ in particular is associated with both drug reward processes and, via functional coupling to NMDA receptors (Conn and Pin, 1997), learning and memory performance (Simonyi et al., 2005). Although selective blockade of mGluR_5_ has been shown to attenuate reinstatement of drug and alcohol seeking (Backstrom et al., 2004; Backstrom and Hyytia, 2006; Gass et al., 2009; Kumaresan et al., 2009; Martin-Fardon et al., 2009; Martin-Fardon and Weiss, 2012), these effects may be compromised in animals with histories of prolonged drug exposure or chemical dependence (Hao et al., 2010; Sidhpura et al., 2010). Antagonism of mGluR_5_ is also associated with deficits in learning and memory tasks (Simonyi et al., 2010), making this strategy an unlikely candidate treatment during extinction training. An alternative approach of using positive allosteric modulators (PAM) of mGluR_5_, which potentiate the receptor response to endogenous glutamate but at moderate doses have no agonist action of their own, has therefore been developed to target extinction learning as a method of establishing long-lasting abstinence from drug-seeking behaviors (Cleva et al., 2010). The systemically active mGluR_5_ PAM 3-cyano-*N*-(1,3-diphenyl-1H-pyrazol-5-yl)benzamide (CDPPB; Kinney et al., 2005) has been recently found to reverse deficits in a novel object recognition task imposed by extended access to METH (Reichel et al., 2011). CDPPB has also been shown to enhance extinction of cocaine-induced conditioned place preference (Gass and Olive, 2009) and reduces cocaine-seeking behavior following intravenous self-administration and deters the reacquisition of cocaine self-administration (Cleva et al., 2011; Nic Dhonnchadha and Kantak, 2011).

This study comprises of experiments designed to test the potential beneficial effect of CDPPB on extinction from responding for METH, as established by a series of self-administration sessions where delivery of METH reinforcement was accompanied by discrete cues. In one experiment, all of the self-administration sessions were restricted to 90 min, and in a parallel experiment the second half of self-administration training comprised of extended (6 h) sessions, permitting elevated daily intake of METH. The effect of CDPPB on extinction learning was also tested in rats trained to self-administer sucrose in the presence of sucrose-associated cues. In all experiments, cue reinstatement testing followed extinction training, and the extinction/reinstatement testing procedure was repeated to ascertain the potential of CDPPB treatment to induce lasting behavioral effects.

## Materials and Methods

### Animals

A total of 56 male Sprague-Dawley rats (Harlan Laboratories, Livermore, CA, USA; 250–275 g upon arrival) were single-housed and maintained on a 12/12 h reversed light/dark cycle, and all training and testing was conducted without food or water restriction during the dark phase of the cycle. All experimental procedures were conducted in accordance to the National Institute of Health Guide for the Care and Use of Laboratory Animals and were approved by the Institutional Care and Use Committee of Arizona State University.

### Experimental design

**Figure 1:**
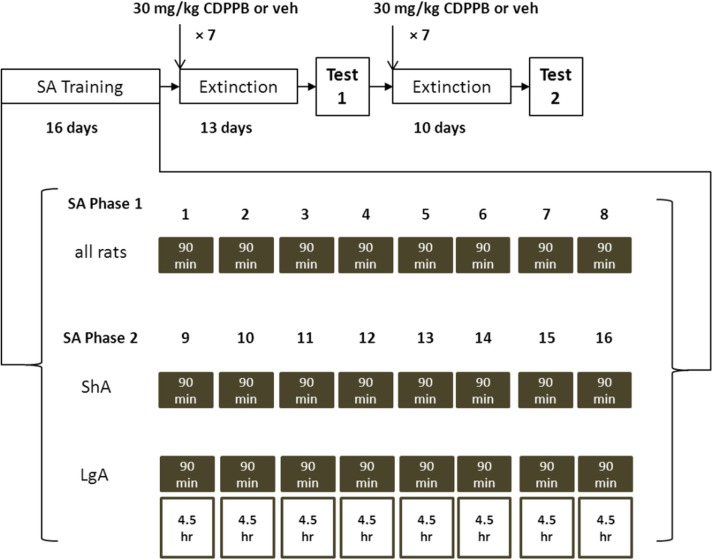
**Block diagram of training and testing regimens used in Experiments 1 and 2**. SA training is divided into two phases: in Phase 1 all rats are trained to SA METH in the presence of lever-contingent sensory cues for 90 min; in Phase 2 ShA rats continue to be trained with cues for 90 min (shaded boxes), and LgA rats are trained in 6-h SA sessions, where the first 90 min continue to deliver METH with cues but the last 4.5 h deliver METH without cues (open boxes). After SA training, rats were subjected to two sequences of daily extinction sessions, the first seven sessions preceded by injections of CDPPB or vehicle. Each extinction sequence was followed by 90-min reinstatement test sessions where rats were re-exposed to METH-paired cues.

In Experiments 1 (initial *n* = 24), and 2 (*n* = 24), rats were trained to self-administer METH over a period of 16 days by pressing a lever, where a combination of visual and auditory cues were presented prior to METH reinforcement (Figure 1). Rats in Experiment 1 were trained using a *short access* (ShA) regimen, where 90-min sessions were used for the entire span of SA training. Rats in Experiment 2 were trained using a *long access* (LgA) regimen, where they were given 90-min sessions for the first 8 days and 6-h sessions for the last 8 days of SA training. The rats were then subjected to extinction training for 13 days, the first seven of which were preceded by injections of CDPPB or vehicle. The post-treatment series of six extinction sessions were included to assess the persistence of treatment effects on extinction responding. Following the initial series of extinction sessions, rats were tested for reinstatement of METH seeking operant behavior triggered by presentation of METH-paired cues. This test was then followed by 10 daily extinction sessions, the first seven of which were also preceded by systemic injections of CDPPB or vehicle, followed by another cue reinstatement test.

In Experiment 3 (*n* = 18), rats were trained to SA sucrose pellets by pressing a lever during 12 daily 30-min sessions. This was followed by 13 days of extinction training, the first seven of which were preceded by injections of CDPPB or vehicle. Following the initial series of extinction sessions, rats were tested for reinstatement of sucrose-seeking operant behavior triggered by presentation of sucrose-paired cues. This test was then followed by 10 daily extinction sessions, the first seven of which were also preceded by systemic injections of CDPPB or vehicle, followed by another cue reinstatement test.

### Surgical procedures

Prior to arrival at the animal facility, 48 of the rats were surgically pre-implanted with Silastic rounded-tip jugular indwelling catheters at Harlan Laboratories, and the catheters were filled with HepLock solution to prevent loss of patency during shipment. Approximately 24 h after arrival, rats were anesthetized with isoflurane (2% v/v, Butler Animal Health Supply, Dublin, OH, USA) vaporized in oxygen at a flow rate of 2 L/min. The rats were also given pre-incision injections of buprenorphine (0.05 mg/kg, s.c., Reckitt Benckiser, Richmond, VA, USA) and meloxicam (1 mg/kg, s.c., Boehringer Ingelheim, St. Joseph, MO, USA). The skin area where the catheter exited between the scapulae was cleaned with 1% iodine (Purdue Products, Stamford, CT, USA). A 2-cm incision was then made to connect the catheter to a back-mounted threaded vascular access port (Plastics One, Roanoke, VA, USA). The catheter was anchored to the port with SNAP dental resin (Parkwell, Edgewood, NY, USA) and secured to the surrounding tissue with a polyethylene mesh collar (Plastics One). The wound was then treated with 0.2 ml bupivacaine hydrochloride (0.25% v/v, Hospira, Lake Forest, IL, USA), closed with nylon sutures (Ethicon, San Lorenzo, Puerto Rico, USA) and topically treated with lidocaine (Hi-Tech Pharmacal, Amityville, NY, USA) and a triple antibiotic gel (G&W Laboratories, South Plainfield, NJ, USA). The HepLock solution was evacuated from the catheter, which was then flushed with 0.2 ml heparin (100 U/ml, Sagent Pharmaceuticals, Schaumberg, IL, USA) and 0.2 ml Timentin (GlaxoSmithKline, Triangle Park, NC, USA). The access port was then sealed with a plastic obturator and a threaded protective cap (Plastics One). Rats were given two injections of 0.9% saline (5 ml each, s.c.) and small portions of “Froot Loops” cereal to facilitate postsurgical rehabilitation. Following surgical procedures, rats were allowed to recover for 5 days. Throughout the experiment, the rats received daily intravenous infusions of 0.2 ml Timentin and 0.2 ml heparin to minimize infections and maintain catheter patency.

### Drugs

Methamphetamine hydrochloride (Sigma Aldrich, St. Louis, MO, USA) was dissolved in 0.9% sterile saline for intravenous (i.v.) self-administration and intraperitoneal (i.p.) injection. CDPPB (3-cyano-*N*-(1,3-diphenyl-1Hpyrazol-5-yl)benzamide; custom synthesized Chemir Analytical, Maryland Heights, MO, USA) was dissolved in 10% v/v Tween 80 (Sigma) and delivered intraperitoneally (i.p.) in a volume of 1 ml/kg, 30 min before behavioral testing.

### Behavioral training and testing

#### Operant methamphetamine self-administration

Starting the day after surgery, rats in Experiments 1 and 2 received 3–5 sucrose pellets (45 mg, TestDiet, Richmond, IN, USA) daily in their home cages. After 5 days of recovery, rats were placed into the operant chambers for two daily 90-min pre-training sessions. Sessions were initiated by extension of an active lever, and thereafter responses on the active lever were reinforced with delivery of a sucrose pellet on a continuous reinforcement schedule. Responses on the inactive lever were registered but resulted in no schedules consequences for the entirety of the experiment. Following pre-training, rats were placed into the operant chambers for daily 90-min self-administration sessions. Each response on the active lever resulted in activation of an infusion pump delivering for 2 s, delivering 0.05 mg/kg METH in 0.06 ml saline. METH reinforcement was accompanied by simultaneous activation of a cue light and tone for 5 s, and a 20 s timeout period during which responses on the active lever had no consequences. In Experiment 1, rats were subjected to the ShA regimen where they received eight additional 90-min training sessions with light and tone cues. In Experiment 2, rats were subjected to the LgA regimen, where they also received eight 90-min sessions with cues, but each session was immediately followed by another 4.5-h session where active lever responses were reinforced with 0.05 mg/kg METH infusions without presentation of cues. This splitting of cue conditions in the LgA sessions was implemented to maintain an equal number of drug-cue exposures experienced by the rats between Experiment 1 and Experiment 2.

Rats in Experiment 3, which were not subjected to surgery but were given sucrose pellets in their home cages for 5 days, were also trained to respond on the active lever for sucrose pellet reinforcement. These training sessions were 30 min and paired pellet delivery with activation of light and tone cues for 5 s and a 20 s timeout period. Sucrose SA training continued for 10 consecutive sessions.

#### Extinction training and pharmacological treatment

Following self-administration training, rats in all experiments were divided into three groups characterized by pharmacological treatment during extinction: CDPPB/veh rats received an injection of 30 mg/kg CDPPB (1 mg/ml, i.p.) 30 min before each of the first seven sessions of the first extinction training series, and an injection of vehicle (10% Tween 80, 1 ml/kg, i.p.) before each of the seven sessions of the second extinction training series; veh/CDPPB rats received vehicle injections before the first seven sessions of the first extinction training series, and CDPPB injections before the first seven sessions of the second extinction training series; veh/veh rats received vehicle injections before the first seven sessions of both extinction training series. During each extinction session, levers were presented but responses on either lever resulted in no programmed consequences. Extinction sessions lasted for 90 min in Experiments 1 and 2 and for 30 min in Experiment 3.

#### Cue-induced reinstatement testing

Reinstatement tests were performed 1 day after the final session of each extinction training series. In Experiments 1 and 2, these tests lasted 90 min under conditions where responses on the active lever resulted in the presentation of METH-paired cues but not METH reinforcement. In Experiment 3, reinstatement test sessions lasted 30 min, where responses on the active lever resulted in presentation of sucrose-paired cues but not delivery of a sucrose pellet. In all experiments, cue reinstatement sessions were not preceded by either METH or CDPPB injections.

#### Statistical analysis

Statistical analyses of data from the three experiments were performed separately. In Experiments 1 and 2, METH reinforcements were analyzed one-way analysis of variance (ANOVA) with *training day* as a within-subjects factor, to assess the change in daily METH intake throughout SA training. Significant effects were followed by direct comparisons between average METH intake of the first eight sessions and second eight sessions of SA training. For Experiment 3, daily responding for sucrose pellets was analyzed by one-way ANOVA with *training day* as a within-subjects factor. Active and inactive lever responses were analyzed by two-way factorial ANOVA, with *lever* (active or inactive) and *training day* as within-subjects factors.

For each experiment, METH reinforcements were also compared between groups identified by their pharmacological treatment during subsequent extinction training, using two-way mixed factorial ANOVAs, with *treatment group* (CDPPB/veh, veh/CDPPB, or veh/veh) as a between-subjects factor and *training day* as a within-subjects factor.

Extinction responses were initially analyzed by 3 × 13 (for the first extinction training sequence) and 3 × 10 (for the second extinction training sequence) mixed factorial ANOVAs, using *treatment group* as a between-subjects factor and *training day* as a within-subjects factor. Significant *treatment group* × *training day* interactions were followed by analyses of the first seven extinction days, corresponding to the sessions preceded by vehicle or CDPPB injections, and the remaining extinction days. These procedures were also mixed factorial ANOVAs (3 × 7 for Days 1–7, and either 3 × 5 or 3 × 3 for the remaining days), using *treatment group* as a between-subjects factor and *training day* as a within-subjects factor.

For all experiments reinstatement behavior were compared against extinction baseline responding (determined by averaging the responding from the final two extinction sessions) using 2 × 3 mixed factorial ANOVAs, using *test condition* (extinction or cues) as a within-subject factor and *treatment group* as a between-subject factor. Significant interactions were followed by *post hoc* two-tailed *t*-tests. Data from first and second reinstatement tests, and data associated with the active and inactive levers, were analyzed separately. All data were presented as mean ± SEM.

## Results

### Experiment 1

#### Methamphetamine training using the ShA regimen

**Figure 2:**
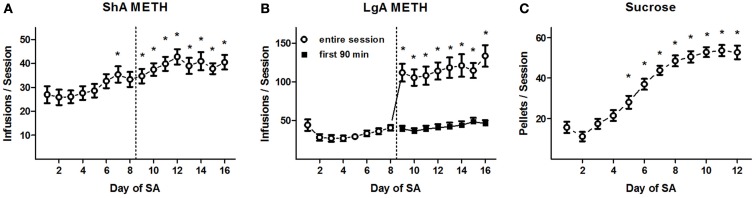
**Reinforcement deliveries during SA training**. **(A)** Mean (±SEM) number of METH reinforcement deliveries per 90-min training session for ShA rats in Experiment 1. *p < 0.05 different from reinforcements delivered during Days 1–3 (Newman–Keuls tests). **(B)** Mean (±SEM) number of METH reinforcement deliveries per 90-min (Days 1–8) or 6-h (Days 9–16, open circles) training session for LgA rats in Experiment 2. Also shown are the mean (±SEM) number of METH reinforcements during the initial 90 min of the 6-h training sessions (Days 9–16, filled circles). *p < 0.05 different from reinforcements delivered during Days 1–8 (Newman–Keuls tests). Dotted line demarcates Phase 1 (left side) and Phase 2 (right side) of METH SA training. **(C)** Mean (±SEM) number of sucrose pellet deliveries per 30-min training session in Experiment 3. *p < 0.05 different from reinforcements delivered during Days 1–3 (Newman–Keuls tests).

Rats trained to SA METH using 16 daily ShA sessions rapidly acquired the behavior and reached a stable baseline of METH reinforcements over the final eight sessions (Figure 2A). ANOVA of the daily METH infusions revealed a significant effect of *training day* (*F*_15, 315_ = 8.9, *p* < 0.0001), and *post hoc* comparisons confirmed that a greater number of reinforcements were delivered during Days 7 and 9–16 (on average, 39.1 ± 1.1 per session) than Days 1–3 (26.3 ± 1.8; Newman–Keuls tests, *p* < 0.05). No other significant *post hoc* differences were found between the amounts of infusions after Day 4, indicating the emergence of a stable baseline of daily METH intake. Additionally, no significant differences in daily METH reinforcements were found between the CDPPB/veh, veh/CDPPB, and veh/veh treatment groups: a main effect of *training day* (*F*_15, 285_ = 9.5, *p* < 0.0001) was found but there was no significant effect of *treatment group* or interaction.

#### Extinction training and the effects of CDPPB treatment

**Figure 3:**
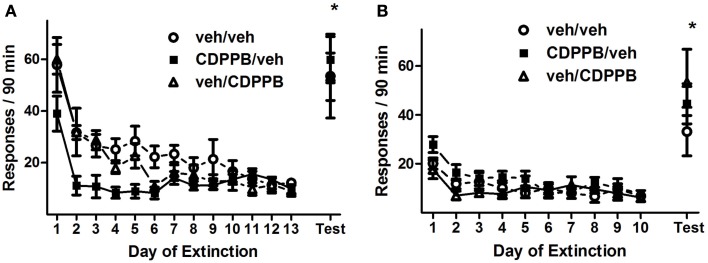
**Active lever responses recorded during extinction training and reinstatement testing in Experiment 1**. **(A)** Mean (±SEM) of active lever responses per 90-min extinction session during the first extinction phase, followed by the initial cue reinstatement test session. **(B)** Mean (±SEM) number of active lever responses per 90-min extinction session during the second extinction phase, followed by the final cue reinstatement test session. Filled symbols indicate pretreatment with 30 mg/kg CDPPB on Days 1–7, and open symbols indicate corresponding vehicle treatment. *p < 0.05 difference between reinstatement testing and average of final two extinction sessions, in all treatment groups (paired *t*-tests).

**Figure A1:**
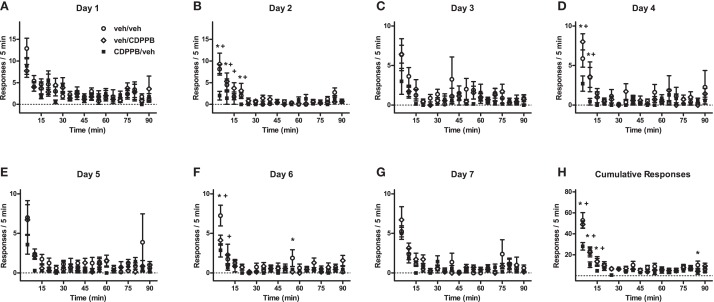
**Time-binned linear analysis of extinction behavior in Experiment 1**. **(A–G)** Mean (±SEM) number of active lever responses from Days 1–7 of the first extinction training sequence, and **(H)** the per-subject average (±SEM) of cumulative responses, which were categorized into 5-min bins and analyzed with mixed factorial ANOVA. *p < 0.05 difference between veh/veh and CDPPB/veh groups (*post hoc* Newman–Keuls tests). +*p* < 0.05 difference between veh/CDPPB and CDPPB/veh groups (Newman–Keuls tests).

Repeated pretreatment injections of CDPPB exerted an attenuating effect on active lever responding during the first series of extinction training sessions, as revealed by a significant *treatment group* × *training day* interaction (Figure 3A; *F*_24, 228_ = 1.6, *p* < 0.05). This effect was isolated to activity during the initial 7 days of extinction training, when the sessions were preceded by injections, as confirmed by the presence of a main effect of *treatment group* in Days 1–7 (*F*_2, 114_ = 1.6, *p* < 0.05) but not in Days 8–13. Additionally, responding on the active lever declined throughout extinction training, as confirmed by main effects of *training day* among all 13 days (*F*_12, 228_ = 19.6, *p* < 0.0001), Days 1–7 (*F*_6, 114_ = 18.8, *p* < 0.0001), and Days 8–13 (*F*_5, 95_ = 2.4, *p* < 0.05). However, CDPPB treatment had no apparent effect on active lever responding during the second series of extinction sessions: a main effect of *training day* was present (*F*_9, 171_ = 12.2, *p* < 0.0001), but no other significant main effects or interactions were found (Figure 3B). Time-binning analysis of the active lever responding revealed significant differences between active lever responding levels of the CDPPB/veh rats and the other groups, which were localized to the beginning time slots (the first 15 min, Figure A1 in Appendix).

#### Reinstatement of METH seeking

The first and second reinstatement tests were similar statistically, in that all groups of rats responded on the active lever to levels that were significantly greater than their extinction baselines, but CDPPB treatment during the preceding sequence of extinction training sessions did not appear to have an effect on responding. Analysis of active lever responding during the first reinstatement test revealed a main effect of *test condition* (*F*_1, 19_ = 68.7, *p* < 0.0001) but no other significant effects or interactions. This was confirmed by the observation that, in all treatment groups, reinstatement activity was at higher levels (veh/veh: 53.6 ± 11.8, CDPPB/veh: 59.9 ± 8.9, veh/CDPPB: 53.3 ± 12.7) than extinction baseline averages (veh/veh: 7.8 ± 2.5, CDPPB/veh: 11.7 ± 1.2, veh/CDPPB: 12.7 ± 5.1; *p* < 0.05, paired *t*-tests).

Similarly, analysis of active lever responding during the second reinstatement test revealed a main effect of *test condition* (*F*_1, 19_ = 38.5, *p* < 0.0001) but no other significant effects or interactions. This was confirmed by the observation that, in all treatment groups, reinstatement activity was at higher levels (veh/veh: 33.1 ± 7.3, CDPPB/veh: 44.6 ± 8.2, veh/CDPPB: 53.3 ± 13.6) than extinction baseline averages (veh/veh: 8.7 ± 0.7, CDPPB/veh: 8.6 ± 0.5, veh/CDPPB: 6.6 ± 0.2; *p* < 0.05, paired *t*-tests). Analysis of inactive lever responding associated with the two reinstatement tests did not reveal significant effects or interactions.

### Experiment 2

#### Methamphetamine training using the LgA regimen

In Experiment 2, the extended conditioning sessions during the second half of SA training were characterized by a dramatic increase on the daily amount of METH consumed (Figure 2B). ANOVA of the daily METH infusions revealed a significant effect of *training day* (*F*_15, 320_ = 22.5, *p* < 0.0001), and *post hoc* comparisons confirmed that a greater number of infusions were exhibited during days 9–16 (115.8 ± 4.2) than days 1–8 (33.2 ± 1.6; Newman–Keuls tests, *p* < 0.05). In contrast, the infusions received during each the first 90 min of each session on Days 9–16 (on average, 42.0 ± 1.6) did not differ significantly from the amount of METH reinforcements received on Days 1–7 (Newman–Keuls tests). No significant *post hoc* differences were revealed in pairwise comparisons among the METH reinforcements received on Days 9–16 (Newman–Keuls tests), suggesting that no significant trend of “escalation” emerged in the time-corrected rate of METH intake as a consequence of the LgA training regimen. Additionally, no significant differences in daily METH reinforcements were found between the CDPPB/veh, veh/CDPPB, and veh/veh treatment groups: a main effect of *training day* (*F*_15, 270_ = 9.5, *p* < 0.0001) was found but there was no significant effect of *treatment group* or interaction.

#### Extinction training and the effects of CDPPB treatment

**Figure 4:**
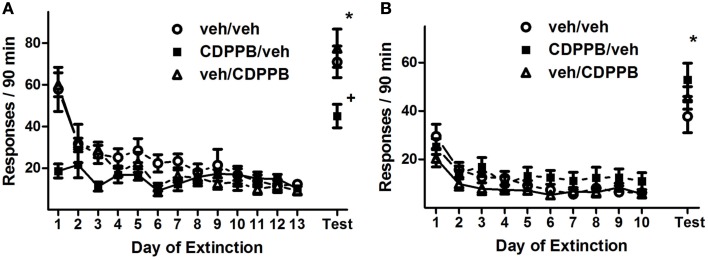
**Active lever responses recorded during extinction training and reinstatement testing in Experiment 2**. **(A)** Mean (±SEM) number of active lever responses per 90-min extinction session during the first extinction phase, followed by the initial cue reinstatement test session. **(B)** Mean (±SEM) number of active lever responses per 90-min extinction session during the second extinction phase, followed by the final cue reinstatement test session. Filled symbols indicate pretreatment with 30 mg/kg CDPPB on Days 1–7, and open symbols indicate corresponding vehicle treatment. *p < 0.05 difference between reinstatement testing and average of final two extinction sessions, in all treatment groups (paired *t*-tests). +*p* < 0.05 difference between CDPPB/veh group and both veh/veh and veh/CDPPB groups (paired *t*-tests).

**Figure A2:**
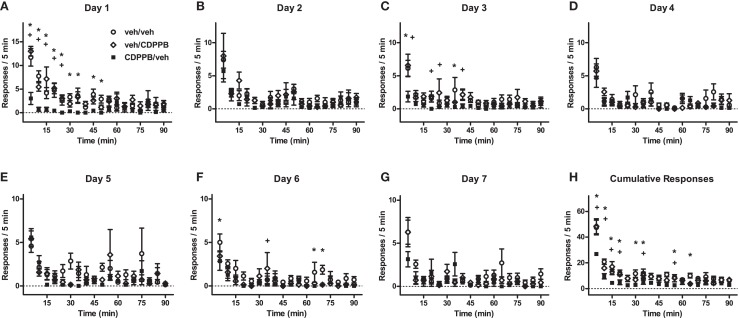
**Time-binned linear analysis of extinction behavior in Experiment 2**. **(A–G)** Mean (±SEM) number of active lever responses from Days 1–7 of the first extinction training sequence, and **(H)** the per-subject average (±SEM) of cumulative responses, which were categorized into 5-min bins and analyzed with mixed factorial ANOVA. *p < 0.05 difference between veh/veh and CDPPB/veh groups (*post hoc* Newman–Keuls tests). +*p* < 0.05 difference between veh/CDPPB and CDPPB/veh groups (Newman–Keuls tests).

Repeated pretreatment injections of CDPPB also had an attenuating effect on active lever responding during the first series of extinction training sessions of Experiment 2, as revealed by a significant *treatment group* × *training day* interaction (Figure 4A; *F*_24, 216_ = 4.6, *p* < 0.0001). This effect was isolated to activity during the initial 7 days of extinction training, when the sessions were preceded by injections, as confirmed by the presence of a significant *treatment group* × *training day* interaction (*F*_12, 108_ = 3.5, *p* < 0.0005) as well as a main effect of *treatment group* (*F*_2, 108_ = 7.7, *p* < 0.005) in Days 1–7, but not in Days 8–13. Additionally, responding on the active lever declined throughout extinction training, as confirmed by main effects of *training day* among all 13 days (*F*_12, 216_ = 23.1, *p* < 0.0001), Days 1–7 (*F*_6, 108_ = 19.2, *p* < 0.0001), and Days 8–13 (*F*_5, 90_ = 6.9, *p* < 0.0005). However, CDPPB treatment had no apparent effect on active lever responding during the second series of extinction sessions: a main effect of *training day* was present (*F*_9, 162_ = 22.3, *p* < 0.0001), but no other significant main effects or interactions were found (Figure 4B). Time-binning analysis of the active lever responding revealed significant differences between active lever responding levels of the CDPPB/veh rats and the other groups, which were mostly restricted to the early time slots (Figure A2 in Appendix).

#### Reinstatement of METH seeking

Analysis of active lever responding during the first reinstatement test revealed a *treatment group* × *test condition* interaction (*F*_2, 18_ = 8.7, *p* < 0.005) as well as a main effect of *test condition* (*F*_2, 18_ = 211, *p* < 0.0001). This was confirmed by the observation that reinstatement activity was lower for the CDPPB/veh group (45.0 ± 5.6) than the other groups (veh/veh: 71.0 ± 7.6, *t*_13_ = 2.7, *p* < 0.05; veh/CDPPB: 77.6 ± 9.1, *t*_13_ = 3.0, *p* < 0.05). However, the reinstatement activity of all groups were higher than their corresponding extinction baseline averages (veh/veh: 10.5 ± 1.3, CDPPB/veh: 12.6 ± 1.5, veh/CDPPB: 9.1 ± 2.2; *p* < 0.05, paired *t*-tests).

In contrast, analysis of active lever responding during the second reinstatement test revealed a main effect of *test condition* (*F*_1, 18_ = 138.4, *p* < 0.0001) but no other significant effects or interactions. This was confirmed by the observation that, in all treatment groups, reinstatement activity was at higher levels (veh/veh: 37.7 ± 6.7, CDPPB/veh: 52.9 ± 6.9, veh/CDPPB: 45.4 ± 4.7) than extinction baseline averages (veh/veh: 6.4 ± 0.9, CDPPB/veh: 11.8 ± 3.5, veh/CDPPB: 7.1 ± 0.9; *p* < 0.05, paired *t*-tests). Analysis of inactive lever responding associated with the two reinstatement tests did not reveal significant effects or interactions.

### Experiment 3

#### Sucrose training

Rats trained to SA sucrose using 12 daily 30-min sessions rapidly acquired the behavior and reached a stable baseline of reinforcements over the final five sessions (Figure 2C). ANOVA of the daily sucrose reinforcements revealed a significant effect of *training day* (*F*_11, 215_ = 36.1, *p* < 0.0001). *Post hoc* comparisons revealed that a greater number of reinforcements were delivered during Days 5–12 (on average, 45.9 ± 1.2) than Days 1–3 (18.8 ± 1.3; Newman–Keuls tests, *p* < 0.05). No other significant *post hoc* differences were found between the amounts of infusions after Day 6, indicating the emergence of a stable baseline of daily sucrose reinforcement. Additionally, no significant differences in daily sucrose reinforcements were found between the CDPPB/veh, veh/CDPPB, and veh/veh treatment groups: a main effect of *training day* (*F*_11, 165_ = 59.3, *p* < 0.0001) was found but there was no significant effect of *treatment group* or interaction.

#### Extinction training and the effects of CDPPB treatment

**Figure 5:**
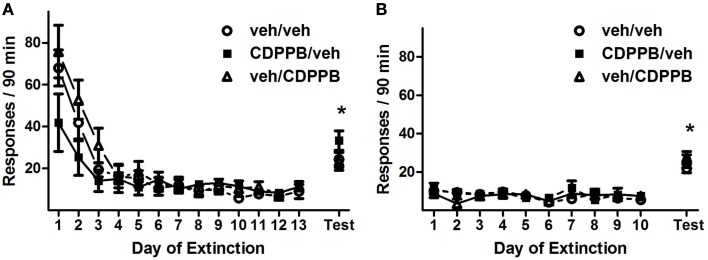
**Active lever responses recorded during extinction training and reinstatement testing in Experiment 3**. **(A)** Mean (±SEM) number of active lever responses per 30-min extinction session during the first extinction phase, followed by the initial cue reinstatement test session. **(B)** Mean (±SEM) number of active lever responses per 30-min extinction session during the second extinction phase, followed by the final cue reinstatement test session. Filled symbols indicate pretreatment with 30 mg/kg CDPPB on Days 1–7, and open symbols indicate corresponding vehicle treatment. *p < 0.05 difference between reinstatement testing and average of final two extinction sessions, in all treatment groups (paired *t*-tests).

**Figure A3:**
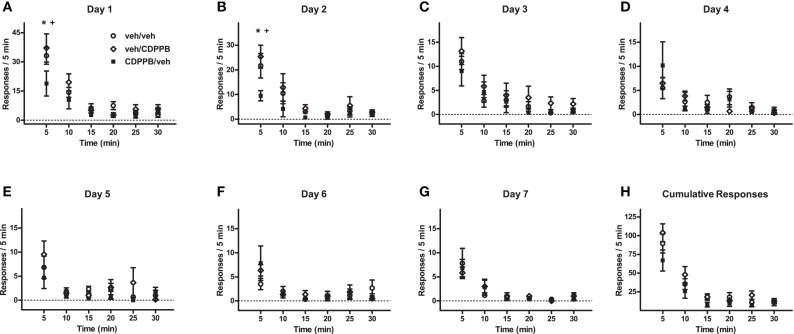
**Time-binned linear analysis of extinction behavior in Experiment 3**. **(A–G)** Mean (±SEM) number of active lever responses from Days 1–7 of the first extinction training sequence, and **(H)** the per-subject average (±SEM) of cumulative responses, which were categorized into 5-min bins and analyzed with mixed factorial ANOVA. *p < 0.05 difference between veh/veh and CDPPB/veh groups (*post hoc* Newman–Keuls tests). +*p* < 0.05 difference between veh/CDPPB and CDPPB/veh groups (Newman–Keuls tests).

Repeated treatment of CDPPB resulted in attenuated responding on the active lever during the first extinction training series of sessions, as revealed by a significant *treatment group* × *training day* interaction (Figure 5A; *F*_24, 180_ = 2.0, *p* < 0.01). This effect was specific to the initial 7 days of extinction training, when the sessions were preceded by injections, as shown by a strong trend toward a significant *treatment group* × *training day* interaction (*F*_12, 90_ = 1.8, *P* = 0.051) in Days 1–7, but no such trends or significant interactions in Days 10–13. Additionally, responding on the active lever declined during the initial 7 days and remained stable for the remainder of extinction training, as revealed by main effects of *training day* among all 13 days (*F*_12, 180_ = 35.2, *p* < 0.0001) and Days 1–7 (*F*_6, 90_ = 36.0, *p* < 0.0001), but not in Days 8–13. As with Experiments 1 and 2, CDPPB treatment had no apparent effect on active lever responding during the second series of extinction sessions: a main effect of *training day* was present (*F*_9, 171_ = 12.2, *p* < 0.0001), but no other significant main effects or interactions were found (Figure 5B). Time-binning analysis of the active lever responding revealed significant differences between active lever responding levels of the CDPPB/veh rats and the other groups, which were exclusively restricted to the first 5 min (Figure A3 in Appendix).

#### Reinstatement of sucrose-seeking

Like Experiments 1 and 2, the rats of Experiment 3 reinstated responding on the active lever during both reinstatement tests. Analysis of active lever responding during the first reinstatement test revealed a main effect of *test condition* (*F*_1, 15_ = 60.0, *p* < 0.0001) but no other significant effects or interactions. This was confirmed by the observation that, in all treatment groups, reinstatement activity was at higher levels (veh/veh: 24.3 ± 3.8, CDPPB/veh: 33.3 ± 4.6, veh/CDPPB: 23.3 ± 4.3) than extinction baseline averages (veh/veh: 7.8 ± 2.5, CDPPB/veh: 9.7 ± 1.5, veh/CDPPB: 9.7 ± 1.0; *p* < 0.05, paired *t*-tests).

Similarly, analysis of active lever responding during the second reinstatement test revealed a main effect of *test condition* (*F*_1, 15_ = 123.7, *p* < 0.0001) but no other significant effects or interactions. This was confirmed by the observation that, in all treatment groups, reinstatement activity was at higher levels (veh/veh: 21.8 ± 2.2, CDPPB/veh: 25.7 ± 3.3, veh/CDPPB: 27.7 ± 2.9) than extinction baseline averages (veh/veh: 5.5 ± 1.6, CDPPB/veh: 7.5 ± 2.0, veh/CDPPB: 7.5 ± 1.3; *p* < 0.05, paired *t*-tests). Analysis of inactive lever responding associated with the two reinstatement tests did not reveal significant effects or interactions.

## Discussion

Repeated injections of CDPPB prior to extinction training sessions resulted in enhanced extinction of operant responding previously associated with METH reinforcement. Rats trained to self-administer METH using ShA and LgA regimens demonstrated reduced active lever responding in the sessions when pre-treated with CDPPB, and the performance differences between treatment groups were generally localized to the early time bins within the extinction sessions, when most of the activity occurred. Like the vehicle-treated groups, the CDPPB-treated rats exhibited the majority of lever responding early in each extinction session, providing evidence that the performance effect of CDPPB was a consequence of an improved learning process, and not a general sedative or locomotor effect. The dose of CDPPB (30 mg/kg) was also lower than another dose (60 mg/kg) recently shown to have minimal effects in an open field locomotor test (Cleva et al., 2011).

However, the reduced levels of active lever responding during extinction learning exhibited by the CDPPB-treated rats could be indicative of either improved learning or an increased sensitivity to non-reinforcement during these sessions. While this theoretical ambiguity cannot be entirely dismissed with the present data, it is noteworthy that the post-treatment extinction sessions were not characterized by any systematic deviation from the low baseline of activity. Additionally, the start of each successive extinction session was separated by 24 h, sufficiently longer than the measured half-life of CDPPB in the Sprague-Dawley rat, 4.4 h (Kinney et al., 2005), in order to avoid direct pharmacological interference with behavior after Day 7 of extinction training.

The effects of CDPPB treatment were not detectable in the sequence of extinction sessions following the first cue reinstatement test, implying that the benefits of this drug were isolated to the early stages of extinction learning. While the levels of operant responding during the second series of extinction sessions were low compared to the first series, both CDPPB treatment (the veh/CDPPB group) and a history of previous CDPPB treatment (the CDPPB/veh group) failed to prevent the initial rise in activity in the first sessions after cue reinstatement in either ShA or LgA METH-trained rats. Furthermore, after extinction training the rats reinstated METH seeking when exposed to cues previously paired with METH reinforcement, regardless of METH exposure, or CDPPB treatment histories. Only LgA rats experiencing cue reinstatement testing for the first time exhibited an attenuation of reinstatement behavior as a consequence of CDPPB treatment, and these subjects still reinstated lever pressing to a level significantly above the extinction baseline of responding. The benefit of CDPPB treatment as given in this set of experiments therefore appears to be restricted to extinction learning taking place the same day as the treatment, and perhaps represents a consolidation of extinction learning. A higher dose of CDPPB, or a more potent mGluR_5_ PAM compound, may be required to exert differences on post-treatment extinction baseline and behavior after an episode of cue-elicited drug-seeking. A previous study demonstrated a clearer difference in cue reinstatement behavior resulting from ShA and LgA METH histories, when rats were treated prior to testing with a ligand of a different subfamily of mGluRs (Kufahl et al., 2012). The greater potential of mGluR_5_ PAM during extinction may therefore be realized when combined with treatments of other mGluRs altered by extensive METH intake (Schwendt et al., 2012).

Both the enhancement of initial extinction learning and absence of subsequent beneficial effects during reinstatement and post-reinstatement extinction training were shared by the animals trained to self-administer sucrose pellets. This observation was not unexpected, given that mice lacking mGluR_5_ have failed to demonstrate extinction of conditioned fear (Xu and Zhu, 2009), and mGluR_5_ enhancement has been shown to improve performance in a variety of spatial learning and memory tasks (Monahan et al., 1989; Ayala et al., 2009; Uslaner et al., 2009).

The presence of enhanced extinction learning performance following systemic 30 mg/kg CDPPB treatment is at variance with a similar study previously performed in our laboratory, which did not find behavioral effects during extinction training after treatment by CDPPB in the same or greater (60 mg/kg) dosages (Widholm et al., 2011). Furthermore, another recent study determined that 60 mg/kg, and not 30 mg/kg, CDPPB was needed to attenuate extinction responding in rats trained to self-administer cocaine (Cleva et al., 2011). Both of these prior studies subjected the rats to the drug-paired cues during extinction training, in order to make the operant behavior more resistant to extinction and hence provide more potential for a treatment-associated contrast (Feltenstein and See, 2006). In addition, the previous METH study used a contextual renewal design to train and test the rats: METH self-administration training took place in one set of operant chambers with a specific set of ambient odor and auditory cues, with lever-contingent cues (A), extinction training then proceeded in a distinct set of chambers with different ambient but matching lever-contingent cues (B), and then renewal of METH seeking was induced by the return to the original context (A) with the METH-paired ambient and lever-contingent cues (Widholm et al., 2011). A pair of Fos protein studies that encompassed a wide range of brain regions revealed that a complex *c-fos* induction pattern induced by exposure to cocaine-paired lever-contingent cues largely, but not completely, overlapped a *c-fos* pattern induced by re-exposure to a cocaine self-administration environment following abstinence (Neisewander et al., 2000; Kufahl et al., 2009). Furthermore, Fos protein expression induced by cocaine-paired cues was absent when the cues had been presented during extinction (Zavala et al., 2007). These results indicated fundamental differences in brain activity in reaction to drug-paired cues, depending on the nature of the cues and their presence or absence during extinction training, which could have implications in the sensitivity of extinction and reinstatement behavior to mGluR_5_ manipulation. Nonetheless, the different results reported by our laboratory under these procedural circumstances underscore the relative fragility of the CDPPB treatment effect found in these data.

In order to equate the number of reinforcer-cue pairings between experiments, rats subjected to the LgA procedure were given METH-paired cues during the first 90 min but not the last 4.5 h of the sessions on Days 9–16 of SA training. The large proportion of METH deliveries in the absence of cues left a possible difference in the predictive value of the light/tone stimulus: the Pavlovian conditioning taking place in the first half of self-administration may be compromised by the subsequent cue-free METH deliveries, but such backward blocking has been known to be very unlikely in rats (Urushihara and Miller, 2010), and the similar interaction effects in the LgA and ShA extinction data suggest that this procedural difference did not interfere with the actions of CDPPB. Moreover, a recent study of extended cocaine self-administration has identified total drug exposure as clearly more important than the proportion of drug-cue pairings in the control of subsequent drug-seeking (Jonkman et al., 2012). Nonetheless, an experiment of this type would need to be performed using METH to fully abrogate the issue of the predictive value of the cue from studies that utilize extended self-administration sessions to create a model of more severe forms of addiction.

Another possible concern arising from the SA training procedures is that the extended period without cues in the LgA sessions created a resemblance between the SA training and extinction sessions that was not matched in the ShA rats. While the LgA training regimen was not a partial reinforcement design, and baseline extinction behavior was not different between ShA and LgA rats in this and a prior set of experiments (Kufahl et al., 2012), it could be argued that the presence of cue-free time within the operant chamber could have translated into a augmented sensitivity to CDPPB treatment. This notion was not overtly supported by the present data, since the moderate effect of treatment specific to LgA rats was not in extinction but the attenuation of cue-triggered reinstatement.

The promising strategy of enhancing extinction learning to counteract compulsive METH seeking was demonstrated by the use of the mGluR_5_ PAM CDPPB. While the learning effect was not long-lasting and not specific to METH, differences in sensitivity in rats with histories of extended METH exposure were revealed in detailed analysis of the early extinction sessions. Comparison of the present results with 30 mg/kg CDPPB and the results with 60 mg/kg we have previously reported reveals the influence of procedural differences that lie within the flexible interpretations of the extinction-reinstatement model. Cognitive enhancement by repeated treatments with an mGluR_5_ PAM appears to be an encouraging avenue of further investigation, using improved methodological regimens.

## Conflict of Interest Statement

The authors declare that the research was conducted in the absence of any commercial or financial relationships that could be construed as a potential conflict of interest.

## Appendix

### Statistical analysis

If significant interactions were found as result of any ANOVAs of the split (Days 1–7 or 8–13) extinction lever responses, the segment of extinction data was then represented in 5-min time bins and analyzed day-by-day using mixed factorial ANOVAs (3 × 18 for Experiments 1 and 2, 3 × 6 for Experiment 3), with *treatment group* as a between-subjects factor and *time* as a within-subjects factor. Significant effects of *treatment group* and significant interactions were followed by between-group Newman–Keuls tests of time-binned responses.

### Results

#### Experiment 1

Time-binned analysis of the active lever responding during Days 1–7 of the first extinction sequence revealed a significant main effect of *time* for all days (Figure A1; 3 × 7 ANOVAs, *p* < 0.05), and *treatment group* × *time* interactions were found in Day 2 (*F*_34, 323_ = 2.5, *p* < 0.0001), Day 4 (*F*_34, 323_ = 1.5, *p* < 0.05), Day 6 (*F*_34, 323_ = 2.1, *p* < 0.0005), and the cumulative responses for Days 1–7 (*F*_34, 323_ = 2.9, *p* < 0.0001). Additionally, a main effect of *treatment group* was found only in Day 2 (*F*_17, 323_ = 3.9, *p* < 0.05). The significant differences between the response levels of the CDPPB/veh rats and the other groups (rats treated with vehicle before the extinction sessions in the first extinction sequence) were primarily localized to the beginning time slots (1–5, 6–10, and 11–15 min, Newman–Keuls tests, *p* < 0.05), with exceptions in Day 2 (16–20 min, *p* < 0.05), Day 6 (50–55 min, *p* < 0.05), and the cumulative responses for Days 1–7 (81–85 min, *p* < 0.05).

#### Experiment 2

Time-binned analysis of the active lever responding during Days 1–7 of the first extinction sequence revealed a significant main effect of *time* for all days (Figure A2; 3 × 7 ANOVAs, *p* < 0.05), but *treatment group* × *time* interactions were found only in Day 1 (*F*_34, 306_ = 3.0, *p* < 0.0001) and the cumulative responses for Days 1–7 (*F*_34, 323_ = 2.9, *p* < 0.0001). However, main effects of *treatment group* were found in Day 1 (*F*_2, 306_ = 13.0, *p* < 0.0005), Day 3 (*F*_2, 306_ = 8.8, *p* < 0.005), Day 6 (*F*_2, 306_ = 5.8, *p* < 0.05), and the cumulative responses for Days 1–7 (*F*_2, 306_ = 7.4, *p* < 0.0001). The significant differences between the response levels of the CDPPB/veh rats and the other groups were more widespread throughout the time bins, compared to Experiment 1: these differences were found in the initial time slot (1–5 min) in Day 1, Day 3, Day 6, and the cumulative responses (Newman–Keuls tests, *p* < 0.05). Further differences in the early time slots were found in Day 1 and the cumulative responses (6–10 and 11–15 min, *p* < 0.05), but not in the other days. In contrast, differences in the later time slots were found in Day 1 (16–20, 21–25, 26–30, 31–35, 41–45, and 46–50 min, *p* < 0.05), Day 3 (16–20, 21–25, 31–35, and 36–40 min, *p* < 0.05), Day 6 (31–35, 61–65, and 66–70, *p* < 0.05), and the cumulative responses for Days 1–7 (16–20, 26–30, 31–35, 51–55, and 61–65 min, *p* < 0.05).

#### Experiment 3

Time-binned analysis of the active lever responding during Days 1–7 of the first extinction sequence revealed a significant main effect of *time* for all days (Figure A3; 3 × 7 ANOVAs, *p* < 0.05), but a trend toward a significant *treatment group* × *time* interaction was found only in Day 1 (*F*_10, 75_ = 1.9, *P* = 0.06), and a significant main effect of treatment group was found only in Day 2 (*F*_2, 75_ = 4.2, *p* < 0.05). The differences between the responses exhibited by the CDPPB/veh rats and those exhibited by the other groups appeared exclusively in the early time bins in Day 1 (1–5 min, Newman–Keuls tests, *p* < 0.05) and Day 2 (1–5 and 6–10 min, *p* < 0.05).
